# Effect of Inadequate Empiric Antibacterial Therapy on Hospital Outcomes in SARS-CoV-2-Positive and -Negative US Patients With a Positive Bacterial Culture: A Multicenter Evaluation From March to November 2020

**DOI:** 10.1093/ofid/ofab232

**Published:** 2021-05-26

**Authors:** Laura Puzniak, Karri A Bauer, Kalvin C Yu, Pamela Moise, Lyn Finelli, Gang Ye, Carisa De Anda, Latha Vankeepuram, Vikas Gupta

**Affiliations:** 1 Merck & Co., Inc., Kenilworth, New Jersey, USA; 2 Becton, Dickinson and Company, Franklin Lakes, New Jersey, USA

**Keywords:** adequate therapy, antimicrobials, bacteria, COVID-19, empiric therapy, SARS-CoV-2

## Abstract

**Background:**

Increased utilization of antimicrobial therapy has been observed during the coronavirus disease 2019 pandemic. We evaluated hospital outcomes based on the adequacy of antibacterial therapy for bacterial pathogens in US patients.

**Methods:**

This multicenter retrospective study included patients with ≥24 hours of inpatient admission, ≥24 hours of antibiotic therapy, and discharge/death from March to November 2020 at 201 US hospitals in the BD Insights Research Database. Included patients had a test for severe acute respiratory syndrome coronavirus 2 (SARS-CoV-2) and a positive bacterial culture (gram-positive or gram-negative). We used generalized linear mixed models to evaluate the impact of inadequate empiric therapy (IET), defined as therapy not active against the identified bacteria or no antimicrobial therapy in the 48 hours following culture, on in-hospital mortality and hospital and intensive care unit length of stay (LOS).

**Results:**

Of 438 888 SARS-CoV-2-tested patients, 39 203 (8.9%) had positive bacterial cultures. Among patients with positive cultures, 9.4% were SARS-CoV-2 positive, 74.4% had a gram-negative pathogen, 25.6% had a gram-positive pathogen, and 44.1% received IET for the bacterial infection. The odds of mortality were 21% higher for IET (odds ratio [OR], 1.21; 95% CI, 1.10–1.33; *P *< .001) compared with adequate empiric therapy. IET was also associated with increased hospital LOS (LOS, 16.1 days; 95% CI, 15.5–16.7 days; vs LOS, 14.5 days; 95% CI, 13.9–15.1 days; *P *< .001). Both mortality and hospital LOS findings remained consistent for SARS-CoV-2-positive and -negative patients.

**Conclusions:**

Bacterial pathogens continue to play an important role in hospital outcomes during the pandemic. Adequate and timely therapeutic management may help ensure better outcomes.

Bacterial superinfection in patients with coronavirus disease 2019 (COVID-19) can complicate clinical management and compromise favorable clinical outcomes [1, 2]. Reported coinfection rates vary widely depending on the population, geographical location, phase of the pandemic, and diagnostic tests used to identify additional pathogens, but are generally low [1–4]. A recent electronic medical record study of 590 960 patients in the United States indicated that almost all (98%) of 17 003 hospitalized severe acute respiratory syndrome coronavirus 2 (SARS-CoV-2)–positive patients had specimens collected for microbiologic testing of additional pathogens. Twenty-one percent of the SARS-CoV-2-positive and SARS-CoV-2-negative patients had positive cultures or diagnostic tests for bacterial, fungal, or non-SARS-CoV-2 viral potential pathogens compared with 28% of patients not tested for SARS-CoV-2 [3]. Although most patients with COVID-19 do not have a detectable non-SARS-CoV-2 pathogen, high rates of empiric antibiotic usage in patients with COVID-19 have been consistently observed (57% to 79% of patients) [2–9], highlighting the challenge of optimizing antimicrobial stewardship practices under complex circumstances. To date, however, the impact of the adequacy of antibacterial therapy on outcomes in patients during the COVID-19 pandemic has not been well characterized.

Recent systematic reviews and meta-analyses from the prepandemic period have supported the association between inadequate empiric therapy (IET) and increased mortality and length of stay (LOS) in patients with bacterial infections [10–12]. Most studies of IET have focused on a narrow group of pathogens, primarily isolated from blood cultures, and sometimes spanning several years [10, 12]. Accordingly, these findings are not easily generalized to the overall usage of antimicrobials in hospitals. In addition, the relevance of such studies to the COVID-19 pandemic, in which patients with SARS-CoV-2 constitute a substantial proportion of the hospitalized population at many institutions, has not been assessed.

We evaluated the impact of IET on clinical outcomes in hospitalized patients with positive bacterial cultures during the COVID-19 pandemic and compared outcomes associated with IET in SARS-CoV-2-positive vs SARS-CoV-2-negative patients. The inclusion of SARS-CoV-2-negative patients allowed us to provide contemporary insights into outcomes associated with IET exclusive of the complexities surrounding SARS-CoV-2 infections.

## METHODS

### Study Design

We conducted a multicenter, retrospective cohort analysis of data from 201 US medical facilities encompassed by the BD Insights Research Database (Becton, Dickinson and Company, Franklin Lakes, NJ, USA), which includes large and small medical care facilities throughout the United States (BD Insights Research Database, Becton, Dickinson & Company, Franklin Lakes, NJ, USA) [13–15]. The primary objective was to evaluate the impact of IET on outcomes (mortality and length of stay) in hospitalized patients with a positive bacterial culture overall and by SARS-CoV-2 testing result.

Eligible admissions were hospitalized adults (≥18 years of age) with a >1-day inpatient admission, ≥24 hours of antibiotic therapy, and a record of discharge or death between March 1, 2020, and November 28, 2020. Study inclusion required a SARS-CoV-2 test; samples for SARS-CoV-2 polymerase chain reaction or antigen testing had to be collected during the period between 7 days before admission and 14 days after admission, consistent with current definitions for non-hospital-onset COVID-19 [16]. A positive culture for gram-negative or gram-positive bacteria with susceptibility results was also required.

Bacterial culture sites included respiratory, blood, urine, skin/wound, intra-abdominal, and other specimens. Microbiology results likely associated with surveillance cultures (eg, nasal or rectal swabs) and environmental cultures were excluded by a previously described methodology that uses source, time of collection, microorganism type, and number of microorganisms in a culture to flag likely contaminated samples [17]. In analyses of outcomes by culture site, patients who had positive specimens from >1 culture site were included in analyses for each site.

The following bacteria with susceptibility results were included: gram-negative bacteria (Enterobacteriaceae [*Escherichia coli*, *Klebsiella pneumoniae*, *Klebsiella oxytoca*, *Enterobacter aerogenes*, *Enterobacter cloacae*, *Serratia marcescens*, *Citrobacter freundii*, *Proteus mirabilis*, and *Morganella morganii*], *Pseudomonas aeruginosa*, *Acinetobacter* species [*A. baumannii* and *A. baumannii/haemolyticus*], and *Stenotrophomonas maltophilia*) and gram-positive bacteria (*Staphylococcus aureus*, *Streptococcus pneumoniae*, *Enterococcus* spp. [*E. faecalis*, *E. faecium*]). Polymicrobial findings were defined as >1 designated gram-positive or gram-negative bacterium obtained from any culture source during the admission.

Empiric antimicrobial therapy with an order for ≥24 hours was evaluated and categorized as adequate or inadequate. IET was defined as (a) antimicrobial therapy within 48 hours from culture collection that did not cover the bacteria and/or to which the bacteria had intermediate susceptibility or resistance or (b) no antimicrobial therapy prescribed within 48 hours from culture collection in a patient with a positive culture. Adequate empiric therapy (AET) was defined as antimicrobial therapy within 48 hours from culture collection that covered the bacteria and to which the bacteria were susceptible.

Outcome and epidemiological studies using this retrospective, deidentified data set were approved, and informed consent waived by the New England Institutional Review Board (Wellesley, MA, USA; IRB No. 120180023).

### Outcomes

We evaluated in-hospital mortality, hospital length of stay (LOS), and intensive care unit (ICU) LOS in patients with adequate or inadequate therapy overall and by SARS-CoV-2 testing result. LOS was based on hospital admission, discharge, and transfer data and calculated as the difference between admission date and discharge date.

Maximum laboratory values recorded within the first 3 days of admission were used as a surrogate for admission period comorbidities, defined as underlying severe illnesses or conditions (Supplementary Table 1) [18]. We evaluated 6 comorbidities: renal insufficiency or failure, liver dysfunction, diabetes, sepsis, suspected heart failure or myocardial inflammation, and cytokine stimulation. Due to lack of timely device data to identify ventilator use, we used the following surrogate definition to imply ventilator use: (a) the patient was started on intravenous (IV)/IV push (IVP) sedation medications (propofol, lorazepam, midazolam, dexmedetomidine, or ketamine) or IV/IVP opioids (fentanyl, remifentanil, sufentanil, hydromorphone) with a duration ≥24 hours AND (b) at least 2 arterial blood gas results collected at least 24 hours apart (on the first day of sedation medication and a subsequent result 24 hours later).

### Statistical Analysis

Baseline characteristics for patients receiving IET vs AET were evaluated by chi-square tests. In the exploratory analysis, we used chi-square tests (Fisher exact tests where appropriate) or *t* tests to evaluate the correlation between each outcome and each of the covariates. In the multivariable analysis, random-intercept logistic regression modeling was used for assessment of mortality, and generalized linear mixed model (GLMM) methods were used for evaluating hospital LOS and ICU LOS with hospital as random effect. The following variables were included in these models: SARS-CoV-2 test result (positive or negative), empiric therapy (AET or IET), discharge month, culture source, age group, sex, SARS-CoV-2 test setting (admission [≤3 days postadmission] or nonadmission [>3 days postadmission]), *Candida albicans* test result (positive or negative), polymicrobial (>1 bacterium) findings, baseline comorbidities, ICU/ventilator criteria, hospital characteristics (bed size, facility type, teaching status), and geographic region based on US Census regions. All analyses were conducted using the Statistical Analysis System (SAS), version 9.4 (SAS Institute, Cary, NC, USA).

## RESULTS

Of 1 976 567 admissions from 201 hospital facilities, 438 888 (22.2%) patients were tested for SARS-CoV-2 and 39 203 (8.9%) patients had a positive bacterial culture. Of these 39 203 patients, 3674 (9.4%) were positive for SARS-CoV-2 and 11 679 (29.8%) were in the ICU and/or met criteria for ventilator support. The mean (SD) age was 66.1 (16.7) years. Most patients (90.1%) were admitted to urban hospitals, and 56.7% of patients were admitted to hospitals with >300 beds (Supplementary Table 2). The largest proportions of patients were admitted to hospitals in the South Atlantic (19.6%) and East South Central (17.7%) regions.

Comorbidities were frequent in this patient population, particularly liver dysfunction (46.5%) and diabetes (43.3%). Overall, 83.7% of patients had 1 of the 6 specified comorbidities. Rates of comorbidities were higher in SARS-CoV-2-positive compared with SARS-CoV-2-negative patients (Table 1).

**Table 1.** Clinical Characteristics by SARS-CoV-2 Status and Adequacy of Antibacterial Empiric Therapy

	SARS-CoV-2 Tested				SARS-CoV-2 Positive				SARS-CoV-2 Negative			
	Total	AET	IET	*P* Value^a^	Subtotal	AET	IET	*P* Value^a^	Subtotal	AET	IET	*P* Value^a^
Pathogen or Characteristic	No. (% of Column)	No. (% of Row)	No. (% of Row)		No. (% of Column)	No. (% of Row)	No. (% of Row)		No. (% of Column)	No. (% of Row)	No. (% of Row)	
All	39 203 (100.0)	21 908 (55.9)	17 295 (44.1)		3674 (100.0)	1922 (52.3)	1752 (47.7)		35 529 (100.0)	19 986 (56.3)	15 543 (43.8)	
Age group, y				<.0001				<.0001				<.0001
<56 (Q1)	9984 (25.5)	4997 (50.1)	4987 (50.0)		835 (22.7)	401 (48.0)	434 (52.0)		9149 (25.8)	4596 (50.2)	4553 (49.8)	
56–68 (median)	9822 (25.1)	5183 (52.8)	4639 (47.2)		959 (26.1)	458 (47.8)	501 (52.2)		8863 (24.9)	4725 (53.3)	4138 (46.7)	
68–79 (Q3)	10 149 (25.9)	5908 (58.2)	4241 (41.8)		980 (26.7)	532 (54.3)	448 (45.7)		9 169 (25.8)	5376 (58.6)	3793 (41.4)	
>79	9248 (23.6)	5820 (62.9)	3428 (37.1)		900 (24.5)	531 (59.0)	369 (41.0)		8348 (23.5)	5289 (63.4)	3059 (36.6)	
Sex				<.0001				<.0001				<.0001
Female	21 072 (53.8)	12 718 (60.4)	8354 (39.7)		2002 (54.5)	1137 (56.8)	865 (43.2)		19 070 (53.7)	11 581 (60.7)	7489 (39.3)	
Male	18 131 (46.2)	9190 (50.7)	8941 (49.3)		1672 (45.5)	785 (47.0)	887 (53.1)		16 459 (46.3)	8405 (51.1)	8054 (48.9)	
ICU admission				<.0001				<.0001				<.0001
Yes	11 221 (28.6)	5839 (52.0)	5382 (48.0)		1590 (43.3)	755 (47.5)	835 (52.5)		9631 (27.1)	5084 (52.8)	4547 (47.2)	
No	27 982 (71.4)	16 069 (57.4)	11 913 (42.6)		2084 (56.7)	1167 (56.0)	917 (44.0)		25 898 (72.9)	14 902 (57.5)	10 996 (42.5)	
ICU or ventilator criteria met				<.0001				<.0001				<.0001
Yes	11 679 (29.8)	6029 (51.6)	5650 (48.4)		1740 (47.4)	815 (46.8)	925 (53.2)		9939 (28.0)	5214 (52.5)	4725 (47.5)	
No	27 524 (70.2)	15 879 (57.7)	11 645 (42.3)		1934 (52.6)	1107 (57.2)	827 (42.8)		25 590 (72.0)	14 772 (57.7)	10 818 (42.3)	
Comorbidity												
Liver dysfunction	18221 (46.5)	9759 (53.6)	8462 (46.4)	<.0001	2196 (59.8)	1079 (49.1)	1117 (50.9)	<.0001	16 025 (45.1)	8680 (54.2)	7345 (45.8)	<.0001
Diabetes	16 960 (43.3)	8928 (52.6)	8032 (47.4)	<.0001	2024 (55.1)	1019 (50.4)	1005 (49.7)	.0082	14 936 (42.0)	7909 (53.0)	7027 (47.1)	<.0001
Heart failure or myocardial inflammation	13 898 (35.5)	7602 (54.7)	6296 (45.3)	.0005	1697 (46.2)	837 (49.3)	860 (50.7)	.0008	12 201 (34.3)	6765 (55.5)	5436 (44.6)	.0267
Sepsis	12 838 (32.7)	7246 (56.4)	5592 (43.6)	.1203	1408 (38.3)	704 (50.0)	704 (50.0)	.0269	11 430 (32.2)	6542 (57.2)	4888 (42.8)	.0101
Renal insufficiency or failure	11 145 (28.4)	5878 (52.7)	5267 (47.3)	<.0001	1371 (37.3)	675 (49.2)	696 (50.8)	.0039	9774 (27.5)	5203 (53.2)	4571 (46.8)	<.0001
Cytokine stimulation	6959 (17.8)	3555 (51.1)	3404 (48.9)	<.0001	1465 (39.9)	710 (48.5)	755 (51.5)	.0001	5494 (15.5)	2845 (51.8)	2649 (48.2)	<.0001
Presence of any of the 6 specified comorbidities				<.0001				.0059				<.0001
Yes	32 818 (83.7)	18 075 (55.1)	14 743 (44.9)		3361 (91.5)	1735 (51.6)	1626 (48.4)		29 457 (82.9)	16 340 (55.5)	13 117 (44.5)	
No	6385 (16.3)	3833 (60.0)	2552 (40.0)		313 (8.5)	187 (59.7)	126 (40.3)		6072 (17.1)	3646 (60.1)	2426 (40.0)	
SARS-CoV-2 test result				<.0001				N/A				N/A
Positive	3674 (9.4)	1922 (52.3)	1752 (47.7)		3674 (100.0)	1922 (52.3)	1752 (47.7)					
Negative	35 529 (90.6)	19 986 (56.3)	15 543 (43.8)						35 529 (100.0)	19 986 (56.3)	15 543 (43.8)	
Bacteria				<.0001				<.0001				<.0001
Gram-positive	10 039 (25.6)	3506 (34.9)	6533 (65.1)		942 (25.6)	288 (30.6)	654 (69.4)		9097 (25.6)	3218 (35.4)	5879 (64.6)	
Gram-negative	29 164 (74.4)	18 402 (63.1)	10 762 (36.9)		2732 (74.4)	1634 (59.8)	1098 (40.2)		26 432 (74.4)	16 768 (63.4)	9664 (36.6)	
Polymicrobial findings				<.0001				.0008				<.0001
Yes	7606 (19.4)	3646 (47.9)	3960 (52.1)		731 (19.9)	342 (46.8)	389 (53.2)		6875 (19.4)	3304 (48.1)	3571 (51.9)	
No	31 597 (80.6)	18 262 (57.8)	13 335 (42.2)		2 943 (80.1)	1580 (53.7)	1363 (46.3)		28 654 (80.6)	16 682 (58.2)	11 972 (41.8)	
Bacteria source				<.0001				<.0001				<.0001
Respiratory	5620 (14.3)	1975 (35.1)	3645 (64.9)		1108 (30.2)	398 (35.9)	710 (64.1)		4512 (12.7)	1577 (35.0)	2935 (65.1)	
Nonrespiratory	33 583 (85.7)	19 933 (59.4)	13 650 (40.7)		2566 (69.8)	1524 (59.4)	1042 (40.6)		31 017 (87.3)	18 409 (59.4)	12 608 (40.7)	
Positive *Candida albicans* test				<.0001				0.0021				<.0001
Yes	829 (2.1)	321 (38.7)	508 (61.3)		152 (4.1)	61 (40.1)	91 (59.9)		677 (1.9)	260 (38.4)	417 (61.6)	
No	38 374 (97.9)	21 587 (56.3)	16 787 (43.8)		3522 (95.9)	1861 (52.8)	1661 (47.2)		34 852 (98.1)	19 726 (56.6)	15 126 (43.4)	

Abbreviations: AET, adequate empiric therapy; ICU, intensive care unit; IET, inadequate empiric therapy; SARS-CoV-2, severe acute respiratory syndrome coronavirus 2.

^a^ *P* values indicate differences in distribution based on AET/IET status.

In the full patient cohort, positive cultures for gram-negative bacteria were about 3-fold more common than positive cultures for gram-positive bacteria (74.4% vs 25.6%) (Table 1). Polymicrobial findings were identified in 19.4% of patients; 11.3% had positive cultures for both gram-positive and gram-negative bacteria. No differences in gram-positive/gram-negative distribution or polymicrobial findings were observed between SARS-CoV-2-positive and -negative patients. The most common overall culture source was urine (52.8%), followed by blood (24.2%), skin (20.4%), respiratory (14.3%), intra-abdominal (1.7%), and other (3.2%; patients could have >1 source) (Supplementary Table 3).

Analysis of baseline characteristics found significant differences in the distribution of most characteristics by IET vs AET status (Table 1). A few notable differences are specifically related to the bacterial infections, including higher usage of IET in patients with gram-positive pathogens (65.1%), polymicrobial findings (52.1%), and respiratory isolates (64.9%).

### Overall Impact of IET

Of 39 203 patients with a positive bacterial culture, 17 295 (44.1%) received IET (Table 2). The absence of empiric treatment in the first 48 hours (12 130; 30.9%) was more common than the use of empiric treatment that was not adequate for the cultured bacteria (5165; 13.2%). In comparisons of IET and AET in observed data, IET was associated with higher mortality (9.1% vs 6.7%), longer hospital LOS (mean, 11.6 vs 9.1 days), and longer ICU LOS (mean, 8.5 vs 6.9 days; *P *< .001 for all outcomes) (Table 2).

**Table 2.** Outcomes by Adequate or Inadequate Empiric Therapy: Observed Data and Univariate Assessment^a^

	Total (n = 39 203)		SARS-CoV-2 Positive (n = 3674)		SARS-CoV-2 Negative (n = 35 529)	
Outcome	AET	IET	AET	IET	AET	IET
No. (%)	21 098 (55.9)	17 295 (44.1)	1922 (52.3)	1752 (47.7)	19 986 (56.3)	15 543 (43.7)
Mortality, No. (%)	1475 (6.7)	1576 (9.1)	433 (22.5)	509 (29.1)	1042 (5.2)	1067 (6.9)
Mean hospital LOS (SD), d	9.1 (9.0)	11.6 (11.0)	14.7 (14.1)	18.0 (15.8)	8.6 (8.2)	10.9 (10.1)
Mean ICU LOS (SD), d	6.9 (8.6)	8.5 (9.9)	14.0 (13.8)	14.8 (13.7)	5.8 (7.0)	7.3 (8.5)

Abbreviations: AET, adequate empiric therapy; ICU, intensive care unit; IET, inadequate empiric therapy; LOS, length of stay; SARS-CoV-2, severe acute respiratory syndrome coronavirus 2.

^a^ *P *< .001 for IET vs AET for all outcomes *except for* SARS-CoV-2-positive ICU LOS (*P* = .265).

Additional factors significantly associated with all 3 outcomes (increased mortality, hospital LOS, and ICU LOS) in univariate analyses included a SARS-CoV-2-positive result, age, male sex, polymicrobial findings, positive respiratory specimens, ICU and/or ventilator criteria met, criteria for any of the 6 specified comorbidities met, geographic region, and hospital characteristics (larger bed size, urban location, and teaching status) (Table 3; Supplementary Table 4). Gram-positive bacteria were associated with increased mortality and hospital LOS (*P *< .001) but did not have a statistically significant association with ICU LOS.

**Table 3.** Clinical Characteristics and Outcomes: Observed Data and Univariate Assessment^a^

	All Patients			ICU Patients	
Pathogen or Characteristic	No. (%)	Mortality, No. (%)	Hospital LOS, Mean (SD), d	No.	LOS, Mean (SD), d
All	39 203 (100)	3051 (7.8)	10.2 (10.0)	11 221	7.7 (9.3)
Age group, y					
<56 (Q1)	9984 (25.5)	568 (5.7)	11.0 (11.5)	2888	9.0 (10.9)
56–68 (median)	9822 (25.1)	824 (8.4)	11.2 (10.8)	3271	8.3 (9.8)
68–79 (Q3)	10 149 (25.9)	893 (8.8)	10.2 (9.3)	3016	7.1 (8.3)
>79	9248 (23.6)	766 (8.3)	8.4 (7.5)	2046	5.5 (6.6)
Sex					
Female	21 072 (53.8)	1403 (6.7)	9.4 (9.4)	5494	6.8 (8.5)
Male	18 131 (46.3)	1648 (9.1)	11.2 (10.6)	5727	8.5 (10.0)
ICU and/or ventilator criteria met	11 679 (29.8)	2395 (20.5)	16.0 (13.5)	11 221	7.7 (9.3)
Comorbidity					
Liver dysfunction	18 221 (46.5)	2359 (13.0)	12.5 (11.6)	6979	9.0 (10.4)
Diabetes	16 960 (43.3)	1944 (11.5)	12.3 (11.6)	6203	8.9 (10.2)
Heart failure or myocardial inflammation	13 898 (35.5)	2039 (14.7)	12.3 (11.5)	6102	8.3 (9.5)
Sepsis	12 838 (32.8)	1981 (15.4)	12.3 (12.0)	5928	8.3 (9.7)
Renal insufficiency or failure	11 145 (28.4)	1860 (16.7)	13.3 (12.1)	4857	8.8 (10.3)
Cytokine stimulation	6959 (17.8)	1208 (17.4)	14.9 (13.8)	3040	11.0 (11.7)
Presence of any of the 6 specified comorbidities					
Yes	32 818 (83.7)	2976 (9.1)	11.0 (10.5)	10 492	7.9 (9.5)
No	6385 (16.3)	75 (1.2)	6.5 (5.7)	729	4.5 (5.7)
SARS-CoV-2 test result					
Positive	3674 (9.4)	942 (25.6)	16.3 (15.0)	1590	14.4 (13.8)
Negative	35 529 (90.6)	2109 (5.9)	9.6 (9.1)	9631	6.5 (7.8)
Bacteria					
Gram-positive	10 039 (25.6)	898 (9.0)	11.0 (9.3)	3021	7.3 (8.2)
Gram-negative	29 164 (74.4)	2153 (7.4)	10.0 (10.3)	8200	7.8 (9.7)
Polymicrobial findings					
Yes	7606 (19.4)	815 (10.7)	14.1 (13.5)	2853	10.0 (11.4)
No	31 597 (80.6)	2 236 (7.1)	9.3 (8.8)	8368	6.9 (8.3)
Bacteria source					
Respiratory	5620 (14.3)	1370 (24.4)	17.4 (14.9)	3821	12.5 (11.7)
Nonrespiratory	33 583 (85.7)	1681 (5.0)	9.0 (8.4)	7400	5.1 (6.5)
Positive *Candida albicans* test					
Yes	829 (2.1)	163 (19.7)	22.3 (17.3)	434	13.7 (13.6)
No	38 374 (97.9)	2888 (7.5)	10.0 (9.6)	10 787	7.4 (9.0)

Abbreviations: ICU, intensive care unit; LOS, length of stay; Q, quartile; SARS-CoV-2, severe acute respiratory syndrome coronavirus 2.

^a^ *P *< .001 for mortality, LOS, and ICU LOS for each characteristic *except for* gram-positive vs gram-negative bacteria (*P *< .001 for mortality and hospital LOS; *P* = .62 for ICU LOS).

**Figure 1.**
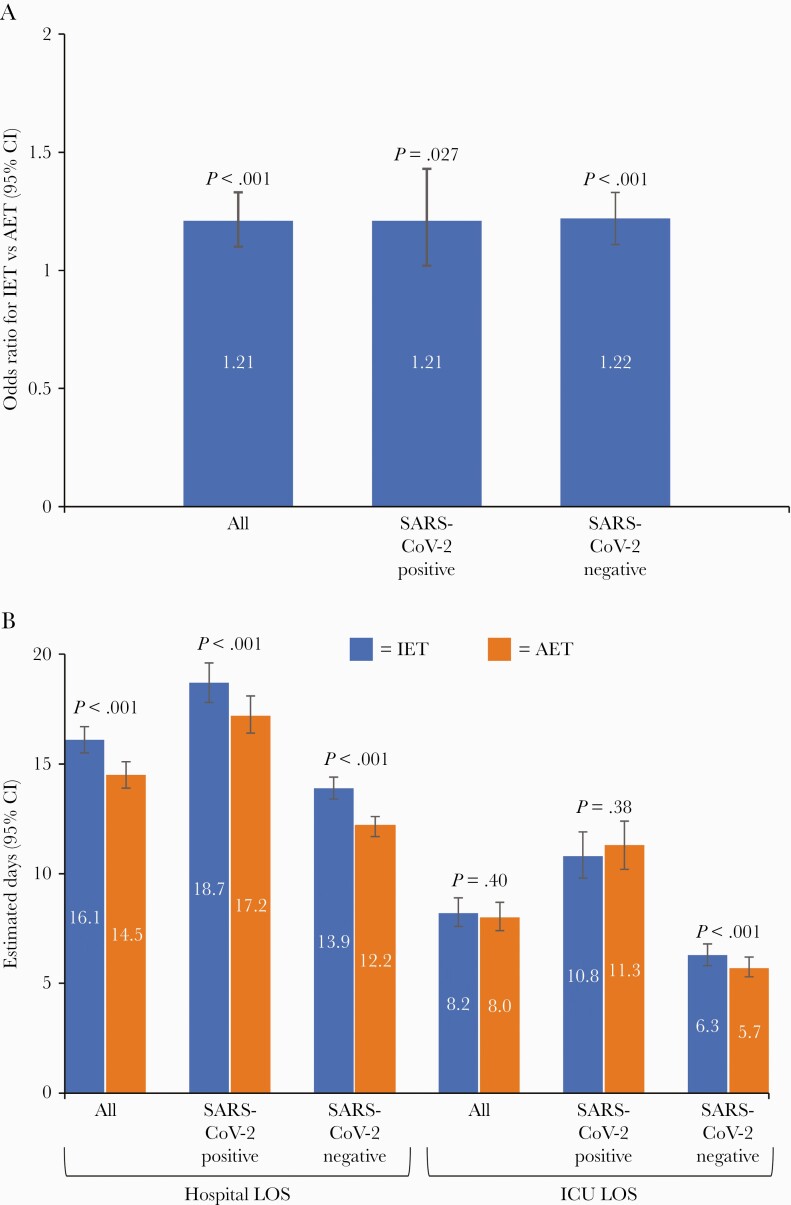
Multivariable model-estimated IET effects on mortality and LOS overall and by SARS-CoV-2 testing status. A, Mortality odds ratios (95% CIs) for IET vs AET. B, Hospital and ICU LOS (estimated days with 95% CI). Abbreviations: AET, adequate empiric therapy; ICU, intensive care unit; IET, inadequate empiric therapy; LOS, length of stay; SARS-CoV-2, severe acute respiratory syndrome coronavirus 2.

In multivariable models, the impact of IET on mortality and hospital LOS was retained (Figure 1; Table 4). IET was associated with a 21% increase in mortality (odds ratio [OR], 1.21; 95% CI, 1.10–1.33; *P *< .001) and a significant increase in hospital LOS compared with AET (estimated difference, 16.1 days; 95% CI, 15.5–16.7 days; vs estimated difference, 14.5 days; 95% CI, 13.9–15.1 days; *P *< .001). The difference in ICU LOS for patients with IET vs AET was not statistically significant (estimated difference, 8.2 days; 95% CI, 7.6–8.9 days; vs estimated difference, 8.0 days; 95% CI, 7.4–8.7 days; *P* = .40) (Figure 1). A SARS-CoV-2-positive result was associated with a ~4-fold increase in mortality in multivariable models as well as significant increases in both hospital and ICU LOS (Table 4).

**Table 4.** Adjusted Effect of IET on Mortality and LOS in Multivariable Models^a^

	Mortality (n = 39 203)		Hospital LOS, d (n = 39 203)		ICU LOS, d (n = 11 221)	
Characteristic	OR (95% CI)	*P value*	Estimated Mean (95% CI)	*P value*	Estimated Mean (95% CI)	*P value*
Empiric therapy		<.001		<.001		.397
AET	Ref		14.5 (13.9–15.1)		8.0 (7.4–8.7)	
IET	1.21 (1.10–1.33)		16.1 (15.5–16.7)		8.2 (7.6–8.9)	
SARS-CoV-2 test result		<.001		<.001		<.001
Negative	Ref		13.0 (12.6–13.5)		6.0 (5.5–6.5)	
Positive	4.04 (3.67–4.45)		18.0 (17.2–18.7)		11.0 (10.1–12.0)	

Abbreviations: AET, adequate empiric therapy; ICU, intensive care unit; IET, inadequate empiric therapy; LOS, length of stay; OR, odds ratio; Ref, reference value; SARS-CoV-2, severe acute respiratory syndrome coronavirus 2.

^a^Other covariates or adjusting variables included discharge month, culture source, age group, sex, *Candida albicans* test status (positive or negative), polymicrobial (yes/no), baseline comorbidities, ICU or ventilator criteria met, hospital characteristics (bed size, facility type, teaching status), and geographic region based on US Census regions. Respiratory source (yes/no) was included in the LOS models but was not included in the mortality model because it did not improve the model fit, despite the fact that this variable significantly affected mortality in univariate assessment. One possible reason is that a highly correlated factor, such as ICU/ventilator status, accounted for part of its effect on mortality. Other reasons, such as confounding from unknown or unobserved factors, may also have played a role in the multivariable modeling analysis.

### Impact of IET by SARS-CoV-2 Test Result

During the time period of this study, 47.7% of SARS-CoV-2-positive and 43.8% of SARS-CoV-2-negative patients with positive bacterial cultures received IET. In observed data and univariate analyses, IET was associated with worse outcomes regardless of patient SARS-CoV-2 result (Table 2). Observed mortality rates in SARS-CoV-2-positive patients were 29.1% for IET vs 22.5% for AET (*P *< .001), and observed mortality rates in SARS-CoV-2-negative patients were 6.9% for IET vs 5.2% for AET (*P *< .001). IET was also associated with significantly longer hospital LOS vs AET in both SARS-CoV-2-positive and -negative patients and with significantly longer ICU LOS in SARS-CoV-2-negative patients (Table 2).

The effect of IET was retained in multivariable-adjusted models (Figure 1). The risk of mortality was increased to a similar extent with IET in both patients positive for SARS-CoV-2 (OR, 1.21; 95% CI, 1.02–1.43; *P* = .027) and those who were negative for SARS-CoV-2 (OR, 1.22; 95% CI, 1.11–1.33; *P *< .001). Compared with AET, IET was associated with significantly longer hospital LOS for both SARS-CoV-2-positive (estimated LOS, 18.7 days; 95% CI, 17.8–19.6 days; vs estimated LOS, 17.2 days; 95% CI, 16.4–18.1 days; *P* = .001) and SARS-CoV-2-negative patients (estimated LOS, 13.9 days; 95% CI, 13.4–14.4 days; vs estimated LOS, 12.2 days; 95% CI, 11.7–12.6 days; *P *< .001). IET was also associated with significantly longer ICU LOS for SARS-CoV-2-negative patients (estimated LOS, 6.3 days; 95% CI, 5.8–6.8 days; vs estimated LOS, 5.7 days; 95% CI, 5.3–6.2 days; *P *< .001), but not SARS-CoV-2-positive patients (estimated LOS, 10.8 days; 95% CI, 9.8–11.9 days; vs estimated LOS, 11.3 days; 95% CI, 10.2–12.4 days; *P* = 0.38) (Figure 1).

## DISCUSSION

In this study of 39 203 hospitalized US adults tested for SARS-CoV-2 with positive cultures for gram-negative or gram-positive bacteria, 44.1% of patients had IET within the 48 hours after culture collection. IET was associated with higher mortality rates and longer LOS compared with AET regardless of SARS-CoV-2 result, but mortality was much higher in SARS-CoV-2-positive patients. Our data indicate that underlying gram-positive and gram-negative bacteria continue to play an important role in hospital outcomes during the COVID-19 pandemic, and one with the potential to be adequately managed to help ensure more favorable outcomes. In addition to evaluating antimicrobial use during the unique time of the pandemic, this study expands the scope of previous IET studies by including multiple bacterial species and culture sites from a large number of hospitalized patients with diverse geographic representation, thereby allowing our findings to be generalized to hospital care throughout the United States. Inclusion of patients who were negative for SARS-CoV-2 allowed us to gain insights into antibiotic therapy patterns in patients being treated for standard conditions. The use of a hospital database allowed us to explore findings in a large, diverse patient population, but as with any database exploration, this study is not definitive. Rather, it should be viewed as a hypothesis-generating analysis that will hopefully help direct further research into this important topic.

Although patients who were positive for SARS-CoV-2 had higher mortality and longer LOS compared with SARS-CoV-2-negative patients, the relative benefit of adequate therapy for the bacterial infection was similar regardless of SARS-CoV-2 result: IET was associated with a ~20% increase in mortality and an additional 1.5 days in hospital LOS in both SARS-CoV-2-positive and -negative patients. In multivariable models, ICU LOS was not significantly associated with IET overall or in SARS-CoV-2-positive patients, possibly due to the impact of multiple comorbid conditions, including organ failure, which may have obscured the effect of IET in the ICU patient subgroup. However, IET was significantly associated with ICU LOS in SARS-CoV-2-negative patients. These patients may provide a truer estimate of the attributable burden of IET on ICU stay without the potentially confounding effect of COVID-19 and its sequelae.

We have shown previously that LOS is ~2-fold higher in SARS-CoV-2-positive or -negative patients with a non-SARS-CoV-2 pathogen (most frequently Enterobacterales spp.) [5]. These data indicate that COVID-19 patients require significant hospital resources, particularly when the admission is complicated by the presence of an additional pathogen. Because we excluded patients with hospital-onset SARS-CoV-2 (positive tests >14 days after hospital admission), our data primarily reflect patients with concomitant or post-SARS-CoV-2 bacterial infections. Despite higher mortality rates, which can shorten the period of hospital resource utilization, the burden of care was greater in SARS-CoV-2-positive patients, as indicated by hospital and ICU LOS, and further increased by IET. It should be noted that the mortality rate of ~25% in SARS-CoV-2 patients observed here, which is comparable to the 23.6% ICU mortality rate for US COVID-19 patients [19], was likely elevated due to the study’s requirement for a positive gram-negative or gram-positive bacterial culture. Other data from our database indicate that the overall COVID-19 mortality rate in all hospitalized patients over this time period was ~11% [18]. High mortality rates were also observed in patients with polymicrobial findings, respiratory specimens as a source of bacteria, a positive *Candida albicans* test, ICU admission or ventilator criteria, and comorbidities at admission.

Consistent with studies of COVID-19 patients [20–22], we found that patients with certain characteristics, including age and male sex, and comorbidities such as renal dysfunction, cardiovascular disease, and cytokine stimulation had higher mortality rates in univariate analyses. The increased mortality rates in patients with any of the 6 specified baseline comorbidities are particularly notable, even within a patient population that was primarily SARS-CoV-2 negative. This elevated risk may be due in part to the use of laboratory surrogates to identify underlying conditions, which could have potentially increased the selection of severely ill patients with acute damage to organ systems, but nevertheless highlights the potential role of comorbidities, which are common in patients with COVID-19 [23], in patient outcomes.

This study was designed so that only patients who received antibiotic therapy for ≥24 hours were included in the analyses. However, 30% of patients with a positive gram-positive or gram-negative bacterial culture did not receive antibacterial therapy within the first 48 hours of culture collection and were therefore categorized as receiving IET. Other studies have reported that delays in antibiotic treatment can negatively impact patient outcomes and costs [11, 24–27]. This study shows that delays are further exacerbated in the era of COVID-19 and could be an important contributor to IET, which is associated with increased mortality and hospital LOS.

In a prepandemic study, the mean laboratory turnaround times for bacterial identification and antimicrobial susceptibility test results in blood specimens were 1.8 and 2.7 days, respectively [28]. Given the significant impact of IET in the first 48 hours of culture collection, reducing delays in achieving effective pathogen-directed treatment remains a critical goal for the care of patients with bacterial infections. Treatment followed by de-escalation as part of an antimicrobial stewardship program may provide a reasonable alternative to delayed therapy [29].

Equally important, however, is the potential overuse of antibiotics in patients who do not have a documented bacterial infection. We and others have found that most patients with SARS-CoV-2 are treated with antibiotics, although bacterial infections are fairly rare [2–9]. Overuse of antibiotics is not unique to the pandemic: An analysis of 2015 data found that the use of antibiotics in patients without a positive urine or blood culture was common [30]. Nevertheless, this trend has been exacerbated by the influx of patients with SARS-CoV-2 infections, who are more likely to receive antibiotics than SARS-CoV-2-negative or -untested patients [5]. Future studies will be required to evaluate whether antimicrobial usage patterns will remain consistent or diminish as clinicians become more accustomed to coping with SARS-CoV-2 infections.

Limitations of the study include the use of institutional facilities as the source of SARS-CoV-2 and bacterial results. There was no central laboratory or uniform method of testing, which may have influenced the results. No case definition for COVID-19 disease was applied, consistent with current medical care practices, so it is possible that some patients with SARS-CoV-2 identified here were asymptomatic but admitted for other causes. Patients with gram-positive or gram-negative pathogens may also have lacked clinically significant infections, although our established algorithm [17] is designed to remove admissions with colonizing microbes from the analyses, and the poorer outcomes in patients receiving IET suggest that positive bacterial cultures were associated with clinically meaningful sequelae. Future analyses are planned to evaluate the impact of IET by culture source. Although we attempted to adjust for relevant factors as identified by our statistical model, it is possible that other factors beyond the scope of our database may have influenced patient outcomes. In particular, LOS and mortality associated with hospital-onset infections may be influenced by the timing of infection and by LOS before infection [31]; these factors were not evaluated in this analysis. Certain geographic areas may have been underrepresented by our database and larger hospitals in more urban areas may have been overrepresented, which could have potentially impacted antimicrobial prescribing practices and testing capabilities.

The data in our study indicate that the high burden of care for hospitalized patients with COVID-19 is further exacerbated by IET in the presence of a non-SARS-CoV-2 pathogen, and that IET is common in hospitalized patients with positive bacterial cultures. Although the ability of IET to negatively impact outcomes is well known [9–11], our findings serve as a valuable reminder that the importance of AET is heightened during times when hospital resources are stretched thin. In addition, our data indicate that IET affects patients with positive bacterial cultures from a number of sources, not just those involving bloodstream infections, which are typically more severe. We hope this study enables stewardship programs to educate and contain IET in hospitals, including the expanded use of rapid diagnostics and initial therapy with agents likely to provide adequate antimicrobial coverage for suspected gram-positive and gram-negative bacteria.

## Supplementary Data

Supplementary materials are available at *Open Forum Infectious Diseases* online. Consisting of data provided by the authors to benefit the reader, the posted materials are not copyedited and are the sole responsibility of the authors, so questions or comments should be addressed to the corresponding author.

ofab232_suppl_Supplementary_Tables
