# Supplementary material for: Effect of Inadequate Empiric Antibacterial Therapy on Hospital Outcomes in SARS-CoV-2-positive and -negative US Patients with a Positive Bacterial Culture: a Multicenter Evaluation from March to November 2020
Source: Open Forum Infect Dis. 2021 May 26:ofab232. doi: 10.1093/ofid/ofab232 (PMC8204877; doi:10.1093/ofid/ofab232)
Supplement: ofab232_suppl_Supplementary_Tables [file ofab232_suppl_supplementary_tables.docx]

**Supplementary Data**

**Supplementary Table 1**. **Laboratory Criteria Used as Surrogates for Admission-period Clinical Conditions.** Values recorded within the first 3 days following admission were considered to indicate the existence of the designated condition.

| **Clinical condition** | **Laboratory criteria** |
| --- | --- |
| Renal insufficiency/failure | Serum creatinine > 2.0 mg/dL |
| Renal failure | Blood urea nitrogen >100 mg/dL AND serum creatinine > 3.0 mg/dL |
| Sepsis | Serum lactate >2.0 mmol/L (sepsis) or >4.0 mmol/L (severe sepsis) |
| Suspected heart failure | Brain natriuretic peptide (BNP) > 400 pg/mL OR N-terminal BNP > 900 pg/mL |
| Myocardial inflammation | 2-fold elevation in troponin drawn within 6 hours and a final value of troponin ≥ 0.4 ng/L, troponin T ≥ 15 ng/L in males and ≥ 10 ng/L in females, or troponin I > 0.04 ng/mL |
| Liver dysfunction | Any of the following: alanine aminotransferase > 60 U/L, aspartate aminotransferase > 80 U/L, serum albumin < 3.0 gm/dL, international normalized ratio >2.0 [and not currently receiving warfarin, rivaroxaban, apixaban, edoxaban, or betrixaban] |
| Cytokine stimulation | Any of the following: fibrinogen < 250 mg/dL, C-reactive protein > 70 mg/L, D-dimer >1000 ng/mL, erythrocyte sedimentation rate > 30 mm/hr, triglycerides > 265 mg/dL |
| Diabetes | Non-insulin diabetes medications prescribed during hospitalization or hemoglobin A1c > 6.5% in the last 6 months |

**Supplementary Table 2**. **Distribution of Facilities and Study Patients by Hospital Characteristics**

| **Characteristics** | **Facilities** | | **Patients** | |
| --- | --- | --- | --- | --- |
|  | **N** | **%** | **N** | **%** |
| Total | 201 | 100 | 39,203 | 100 |
| Rural/urban |  |  |  |  |
| Rural | 55 | 27.4 | 3876 | 9.9 |
| Urban | 146 | 72.6 | 35327 | 90.1 |
| Bed size |  |  |  |  |
| <100 | 68 | 33.8 | 2858 | 7.3 |
| 100-300 | 80 | 39.8 | 14131 | 36.1 |
| >300 | 53 | 26.4 | 22214 | 56.7 |
| Teaching status |  |  |  |  |
| Non-teaching | 143 | 71.1 | 19112 | 48.8 |
| Teaching | 58 | 28.9 | 20091 | 51.3 |
| US census region (states) |  |  |  |  |
| West North Central  (IA, KS, MN, MO, ND, NE, SD) | 4 | 2.0 | 85 | 0.22 |
| New England  (CT, MA, ME, NH, RI, VT) | 3 | 1.5 | 813 | 2.1 |
| Mountain  (AZ, CO, ID, MT, NM, NV, UT, WY) | 8 | 4.0 | 829 | 2.1 |
| East North Central  (IL, IN, MI, OH, WI) | 30 | 14.9 | 5273 | 13.5 |
| Pacific  (AK, CA, OR, WA) | 19 | 9.5 | 5520 | 14.1 |
| Middle Atlantic  (NJ, NY, PA) | 26 | 12.9 | 5567 | 14.2 |
| West South Central  (AR, LA, OK, TX) | 47 | 23.4 | 6501 | 16.6 |
| East South Central  (AL, KY, MS, TN) | 30 | 14.9 | 6944 | 17.7 |
| South Atlantic  (DE, DC, FL, GA, MD, NC, SC, VA, WV) | 34 | 16.9 | 7671 | 19.6 |

**Supplementary Table 3. Source Distribution for Bacterial Cultures and Association with Mortality and LOS: Observed Data and Univariate Assessment**

| **Source** | **All patients** | | | | | **ICU patients** | |  |
| --- | --- | --- | --- | --- | --- | --- | --- | --- |
|  | **n (%)** | **Mortality**  **n (%)** | ***P value*** | **Hospital LOS**  **Mean (SD) days** | ***P value*** | **n** | **LOS**  **Mean (SD) days** | ***P value*** |
| All | 39, 203 (100) | 3,051 (7.8) |  | 10.2 (10.0) |  | 11,221 | 7.7 (9.3) |  |
| Urine |  |  | <.0001 |  | <.0001 |  |  | <.0001 |
| No | 18,516 (47.2) | 1,841 (9.9) |  | 12.1 (11.1) |  | 6,502 | 9.1 (10.3) |  |
| Yes | 20,687 (52.8) | 1,210 (5.9) |  | 8.9 (8.6) |  | 4,719 | 5.6 (7.2) |  |
| Blood |  |  | .0007 |  | <.0001 |  |  | <.0001 |
| No | 29,699 (75.8) | 2,234 (7.5) |  | 10.1 (10.0) |  | 8,123 | 8.0 (9.3) |  |
| Yes | 9,504 (24.2) | 817 (8.6) |  | 10.8 (10.2) |  | 3,098 | 6.8 (9.3) |  |
| Skin |  |  | <.0001 |  | <.0001 |  |  | <.0001 |
| No | 31,210 (79.6) | 2,791 (8.9) |  | 10.0 (10.0) |  | 9,672 | 7.8 (9.4) |  |
| Yes | 7,993 (20.4) | 260 (3.3) |  | 11.2 (10.0) |  | 1,549 | 6.6 (8.7) |  |
| Respiratory |  |  | <.0001 |  | <.0001 |  |  | <.0001 |
| No | 33,583 (85.7) | 1,681 (5.0) |  | 9.0 (8.4) |  | 7,400 | 5.1 (6.5) |  |
| Yes | 5,620 (14.3) | 1,370 (24.4) |  | 17.4 (14.9) |  | 3,821 | 12.5 (11.7) |  |
| Intra-abdominal |  |  | .06 |  | <.0001 |  |  | .83 |
| No | 38,457 (98.3) | 3013 (7.8) |  | 10.2 (10.0) |  | 10,960 | 7.7 (9.3) |  |
| Yes | 656 (1.7) | 38 (5.6) |  | 13.4 (12.3) |  | 261 | 7.4 (9.3) |  |
| Other |  |  | .43 |  | <.0001 |  |  | .21 |
| No | 37,952 (96.8) | 2,961 (7.8) |  | 10.2 (10.0) |  | 10,776 | 7.6 (9.3) |  |
| Yes | 1,251 (3.2) | 90 (7.2) |  | 12.6 (11.0) |  | 445 | 8.2 (9.9) |  |

**Supplementary Table 4**. **Hospital Characteristics, Geographic Regions, and Additional Characteristics Associated with Mortality and LOS: Observed Data and Univariate Assessment**

| **Pathogen or characteristic** | **All patients** | | | **ICU patients** | |
| --- | --- | --- | --- | --- | --- |
|  | **n (%)** | **Mortality**  **n (%)** | **Hospital LOS**  **Mean (SD) days** | **n** | **LOS**  **Mean (SD) days** |
| All | 39, 203 (100) | 3,051 (7.8) | 10.2 (10.0) | 11,221 | 7.7 (9.3) |
| Bed size^a^ |  |  |  |  |  |
| 1-100 | 2,858 (7.3) | 177 (6.2) | 7.7 (7.2) | 741 | 5.9 (6.7) |
| 100-300 | 14,131 (36.1) | 900 (6.4) | 8.8 (8.1) | 3,627 | 6.7 (8.6) |
| 300+ | 22,214 (56.7) | 1,974 (8.9) | 11.5 (11.2) | 6,853 | 8.3 (9.8) |
| Facility type^a^ |  |  |  |  |  |
| Urban | 35,327 (90.1) | 2,816 (8.0) | 10.5 (10.3) | 10,368 | 7.9 (9.5) |
| Rural | 3,876 (9.9) | 235 (6.1) | 8.0 (7.2) | 853 | 4.7 (4.9) |
| Teaching status^a^ |  |  |  |  |  |
| Teaching | 20,091 (51.3) | 1,809 (9.0) | 11.4 (11.1) | 6,266 | 8.2 (9.8) |
| Non-teaching | 19,112 (48.8) | 1,242 (6.5) | 9.0 (8.6) | 4,955 | 6.9 (8.5) |
| Admitted within prior 90 days^b^ |  |  |  |  |  |
| No | 28,942 (73.8) | 2,279 (7.9) | 10.1 (10.1) | 8,431 | 8.1 (9.8) |
| Yes | 10,261 (26.2) | 772 (7.5) | 10.5 (9.8) | 2,790 | 6.2 (7.5) |
| SARS-CoV-2 test setting^a^ |  |  |  |  |  |
| Admission | 35,010 (89.3) | 2,784 (8.0) | 9.9 (9.9) | 9,989 | 7.5 (9.2) |
| Non-admission | 4,193 (10.7) | 267 (6.4) | 13.2 (10.7) | 1,232 | 8.6 (10.1) |
| US census region^a^ |  |  |  |  |  |
| South Atlantic | 7,671 (19.6) | 546 (7.1) | 10.3 (9.4) | 2,055 | 8.1 (9.1) |
| East South Central | 6,944 (17.7) | 561 (8.1) | 11.9 (11.6) | 2,181 | 9.0 (9.9) |
| West South Central | 6,501 (16.6) | 575 (8.8) | 9.8 (9.4) | 1,968 | 7.7 (10.2) |
| Middle Atlantic | 5,567 (14.2) | 407 (7.3) | 10.0 (9.6) | 1,638 | 6.7 (8.0) |
| Pacific | 5,520 (14.1) | 441 (8.0) | 9.6 (10.1) | 1,517 | 6.7 (8.6) |
| East North Central | 5,273 (13.5) | 371 (7.0) | 9.7 (9.8) | 1,290 | 6.9 (8.9) |
| Mountain | 829 (2.1) | 56 (6.8) | 8.8 (6.7) | 256 | 6.0 (6.9) |
| New England | 813 (2.1) | 86 (10.6) | 10.1 (11.3) | 276 | 9.2 (12.5) |
| West North Central | 85 (0.2) | 8 (9.4) | 7.2 (5.6) | 40 | 5.8 (6.8) |
| Discharge month^a^ |  |  |  |  |  |
| 3 | 290 (0.7) | 24 (8.3) | 6.2 (3.8) | 101 | 3.9 (3.3) |
| 4 | 2,380 (6.1) | 319 (13.4) | 8.8 (6.5) | 828 | 5.8 (6.2) |
| 5 | 4,123 (10.5) | 357 (8.7) | 10.2 (8.8) | 1,241 | 7.4 (9.0) |
| 6 | 5,080 (13.0) | 358 (7.1) | 10.8 (10.5) | 1,524 | 7.7 (9.1) |
| 7 | 5,650 (14.4) | 411 (7.3) | 10.6 (10.7) | 1,587 | 8.1 (9.6) |
| 8 | 5,747 (14.7) | 431 (7.5) | 10.4 (10.2) | 1,595 | 7.9 (9.2) |
| 9 | 5,471 (14.0) | 372 (6.8) | 10.5 (11.1) | 1,504 | 8.0 (9.7) |
| 10 | 5,586 (14.3) | 370 (6.6) | 9.8 (9.5) | 1,528 | 7.8 (9.8) |
| 11 | 4,876 (12.4) | 409 (8.4) | 10.3 (10.3) | 1,313 | 8.1 (10.2) |

^a^*P* < .001 for mortality, hospital LOC, and ICU LOS

^b^*P* = .25 for mortality, .002 for hospital LOS, and <.001 for ICU LOS

Abbreviations: ICU, intensive care unit; LOS, length of stay; Q, quartile; SARS-CoV-2, severe acute respiratory syndrome coronavirus 2; SD, standard deviation.
